# The Role of the Anion Insertion‐Extraction Reaction in Amorphous Carbon Thin Film Electrodes on the Vanadium(IV/V) Reaction Probed by Scanning Electrochemical Cell Microscopy

**DOI:** 10.1002/smll.202507044

**Published:** 2025-10-07

**Authors:** Maximilian Hamann, Jens Carthäuser, Diana Rata, Nico Remmler, Michael Bron, Matthias Steimecke

**Affiliations:** ^1^ Martin‐Luther‐Universität Halle‐Wittenberg Institut für Chemie, Technische Chemie I Von‐Danckelmann‐Platz 4 06120 Halle/Saale Germany; ^2^ Martin‐Luther‐Universität Halle‐Wittenberg Institut für Physik Von‐Danckelmann‐Platz 3 06120 Halle/Saale Germany

**Keywords:** all‐vanadium redox flow battery, carbon degradation, fluorescence, microelectrochemistry, N‐doped carbon, raman microscopy, scanning electrochemical cell microscopy

## Abstract

The influence of high potentials on amorphous nitrogen‐free and nitrogen‐doped hydrogenated carbon thin film electrodes with thicknesses of 9 to 30 nm is probed toward the vanadium(IV/V) redox reaction by scanning electrochemical cell microscopy (SECCM), which mimics the reaction of the positive side of the all‐vanadium redox flow battery (VRFB). Besides the evaluation of the peak separation (E_PP_) from cyclic voltammograms (CV), the localized probing is adapted in a way that the influence of high overpotentials on the stability of the carbon materials, as well as competitive electrochemical processes, can be analyzed. The sulfate anion insertion process is found to be the predominant process in all samples, with its onset appearing in parallel to the vanadium(IV/V) reaction. The presence of pyridine/pyrrole groups can stabilize the insertion compound, which inhibits the vanadium(IV/V) reaction much more strongly. In all cases, the electrochemical redox features of the vanadium(IV/V) reaction, as well as the initial Raman spectra of the carbon thin films, are fully reconstructed by applying reductive potentials in a suitable time frame, even after polarizing to drastically high potentials (2.5 V vs. RHE). Overall, this competing insertion reaction must be given greater consideration when discussing electrochemical data of the vanadium(IV/V) redox reaction.

## Graphical abstract

This study examines how high potentials affect amorphous (nitrogen‐doped) carbon thin film electrodes in vanadium(IV/V) redox reactions, relevant for the all‐vanadium redox flow battery. Using electrochemical microscopy, the research highlights that sulfate anion insertion predominates and inhibits vanadium redox activity, especially with pyridine‐ and pyrrole‐like moieties. Despite high polarization, the electrodes’ properties could be fully restored by suitable reductive potentials.

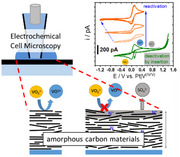

## 1 Introduction

Carbon materials represent an important class of materials in electrochemical energy conversion, whether as support for the active component or as the active material itself. A particularly important example of this is the vanadium redox flow battery (VRFB), which is one of the most promising technologies for the sustainable and economical long‐term storage of regeneratively produced electricity.^[^ ^1^, ^2^ ^]^ It is the most advanced and commercialized flow battery system^[^ ^3^ ^]^ and is characterized by the energy carriers in the aqueous liquid electrolytes, which are stored in tanks and pumped through an electrochemical conversion unit. Consequently, energy and power can be scaled independently and adjusted as required. The uniqueness of the system results from the homogeneous redox pairs of the four different oxidation states of vanadium (negative half‐cell: V^2+^/V^3+^, positive half‐cell: V^(IV)^O^2+^/V^(V)^O_2_^+^), which can be fully regenerated even in the case of partial mixing, e.g., by cross‐contamination through the membrane.

Understanding the effects and processes at the VRFB electrodes, as well as stabilityissues, is the latest aspects that research is addressing.^[^ ^4^ ^]^ The overarching goal is to design well‐defined carbon materials to affect the reaction kinetics and long‐term stability in a suitable way.^[^ ^5^, ^6^ ^]^ Therefore, the understanding of the reaction pathways, the selectivity, and mechanisms, as well as the interaction with the sulfuric acid‐based electrolyte, is of particular interest.^[^ ^7^ ^]^ In the case of the negative side of the VRFB, recently, an in situ‐formed Cu@Cu_6_Sn_5_ core‐shell catalyst on top of a graphite felt electrode was suggested, which accelerates the reaction kinetics and selectively inhibits the hydrogen evolution reaction (HER).^[^ ^8^ ^]^ Besides selectivity issues, a possible presence of various vanadium compounds in the reaction mixture may influence the electrochemical properties of the carbon electrode material. Here, the processes at the positive side of the VRFB are often emphasized as high potentials, and the corrosive electrolyte can strongly affect the carbon material. In addition to the expected VO^2+^ and VO_2_^+^ ions, other monomers and dimers have to be considered, especially in combination with the other components of the electrolyte solution (H_2_O, SO_4_^2−^, HSO_4_^−^).^[^ ^9^ ^]^ Several studies suggest that these compounds (e.g., V_2_O_3_^3+^ or VO_2_SO_4_^‐^), partially formed parallel to the desired reactions, may form an adsorbate layer depending on the conditions (e.g., electrolyte composition, time, applied overpotential, and others), which covers the electrode surface.^[^ ^10^, ^11^, ^12^, ^13^, ^14^ ^]^ Regarding the vanadium(IV/V) redox pair, these adsorbates (VO_x_) particularly influence the vanadium(V) reduction, which is significantly more inhibited compared to the oxidation. Gattrell et al. showed that oxygen‐containing surface groups on the electrode, in combination with adsorption phenomena, make a significant contribution to it.^[^ ^10^ ^]^ Besides the possible formation of a passivating layer at high potentials, the role of sulfate insertion, which is intercalation in the case of graphite materials, as a parasitic process at high sulfuric acid concentration was already addressed in early work.^[^ ^15^ ^]^ In the case of graphite, the electrochemical formation of a graphite intercalation compound (GIC) is well understood by studying the phenomenon with X‐ray diffraction and Raman spectroscopy techniques.^[^ ^16^, ^17^ ^]^ Only little attention is paid to amorphous carbons, which may be due to the fact that the aforementioned methods cannot provide any information from carbon in a disordered state. However, anion insertion and the consecutive processes, like exfoliation by chemical and electrochemical treatment, can be found when using disordered carbon materials.^[^ ^18^ ^]^ In addition to pure carbon electrodes, the controlled introduction of (surface) heteroatoms and their influence on the vanadium redox reaction are also within the focus of research. In the case of oxygen containing surface groups, its influence is discussed controversially.^[^ ^19^ ^]^ The introduction of nitrogen (N‐doping), however, was found to be positive from pyridinic/pyrrolic nitrogen on the reaction rate at 3D graphite felt^[^ ^20^ ^]^ and at planar amorphous carbon electrodes.^[^ ^21^ ^]^

Scanning Electrochemical Cell Microscopy (SECCM), which is a highly relevant type of the microelectrochemical (µEC) or small‐area techniques,^[^ ^22^ ^]^ has gained significant importance in recent years and has already been used to analyze carbon substrates, such as graphene,^[^ ^23^ ^]^ amorphous, and N‐doped amorphous carbon samples.^[^ ^24^ ^]^ In combination with vanadium, SECCM was applied to study pseudocapacitance of V_2_O_5_ nanoparticles^[^ ^25^ ^]^ and also to study kinetics of the positive half‐cell reaction of the VRFB at N‐doped graphene oxide flakes.^[^ ^26^ ^]^

In this work, a SECCM method is used to characterize 9 to 30 nm carbon thin film electrodes by cyclic voltammetry (CV) under VRF battery‐like in situ conditions. Degradation and related phenomena of the carbon associated with the vanadium(IV/V) reaction are studied by a subsequent increase of the upper vertex potential of the CV probing at a fresh sample position, respectively. Raman microscopy is used afterward to gain further insights into structural changes of the thin films at the probed positions. Finally, the influence of N‐doping toward the observed competitive processes is evaluated, and a model is suggested.

## 2 Results and Discussion

Carbon thin films can be prepared by an adapted method of ambient pressure chemical vapor deposition (APCVD)^[^ ^29^ ^]^ on quartz glass at high temperatures in the presence of hydrogen and a carbon source. In this work, cyclohexane and acetonitrile were used as organic precursors; both are already known for the synthesis of carbon^[^ ^30^ ^]^ and N‐doped^[^ ^31^ ^]^ carbon films on glass substrates, respectively. Different film thicknesses were achieved by varying the contact time of the respective organic precursor in the gas flow to the substrate. Depending on the thickness, the samples showed greyish or mirror‐like surfaces and were (semi)‐transparent (Figure S1a,b, Supporting Information). SEM micrographs of the samples with increased layer thickness showed a layered structure of the carbon and N‐doped carbon thin films (Figure S1c,d, Supporting Information). Numerous samples were synthesized as described before, and the thickness of the carbon film on top of the quartz substrate was determined by X‐ray reflectivity (XRR) measurements. For details, see Figure S1e (Supporting Information). Additionally, the sheet resistance was measured using a four‐point‐probe method according to Valdes^[^ ^32^ ^]^ (see Figure S1f, Supporting Information). Furthermore, X‐ray diffraction (XRD) was used to analyze the bulk structure of the films on the quartz substrate. Representative diffractograms of both types of samples (from cyclohexane – a‐C, from acetonitrile – N‐a‐C) show a broad signal between 15 and 30° 2θ, indicating an amorphous carbon structure (see Figure S2a, Supporting Information). Quartz substrates can further be used to perform infrared (IR) spectroscopy in a limited spectral region (2000–4000 cm^−1^). Transmission spectra (Figure S2b, Supporting Information) show the characteristic signals between 3000 and 2800 cm^−1^, which are caused by various CH stretching modes from C‐H moieties in both types of films, as expected from the decomposition of the hydrocarbons.^[^ ^33^ ^]^ In case of the samples synthesized from the acetonitrile precursor, no evidence of remaining nitrile groups can be found in the IR spectrum (≈2220 cm^−1^).

**Figure 1:**
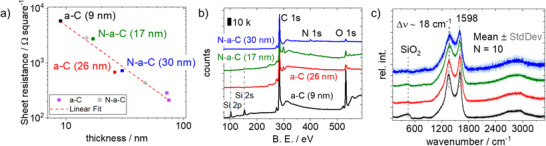
a) Sheet resistance plotted against the carbon or N‐doped carbon thin layer thickness on the quartz glass substrate, b) XPS survey spectrum of four selected samples (B.E. – binding energy), and c) mean Raman spectra of ten single measurements of the same four selected samples. The color code of b) is also valid in c).

The results of the thickness and sheet resistance measurements of eight samples are summarized in **Figure** **1**a. As expected, the sheet resistance increases with decreased layer thickness. From these results, four samples were selected for more in‐depth structural characterization and for later microelectrochemical analysis, two without (a‐C) and two with N‐doping (N‐a‐C). In both cases, different layer thicknesses were chosen, too. To get information about the surface topography and the structure of the selected samples, electron microscopy images can be found in Figure S3 (Supporting Information). All samples exhibit a grain structure consisting of amorphous agglomerates with nanostructured surfaces of comparable dimensions. In the case of the a‐C (9 nm) sample, these amorphous grains appear slightly smaller. To gain more insights of the surface composition, the four samples were further characterized by X‐ray photoelectron spectroscopy (XPS). The survey scans can be found in Figure 1b. In all samples, carbon (C 1s, 284 eV) and oxygen (O 1s, 532 eV) are present. In case of the N‐a‐C samples, nitrogen (N 1s, 400 eV) is additionally present, which confirms a successful incorporation of nitrogen during high temperature synthesis. Furthermore, both thinner samples show the presence of silicon (Si 2s and 2p), which is of high intensity at the 9 nm a‐C sample. This is an expected result of the very low carbon layer thickness and the underlying quartz glass substrate, which contributes to the spectrum at both Si 1s, 2p, and O 1s regions. For this reason, quantitative comparisons of atomic ratios are neglected for the thinner samples. In case of the a‐C (26 nm) and the N‐a‐C (30 nm) samples, a composition of carbon (95.3 at.%), oxygen (4.7 at.%), and carbon (93.5 at.%), oxygen (2.9 at.%), and nitrogen (3.6 at.%) was found, respectively. As suggested from IR measurements (Figure S2b, Supporting Information), an additional contribution of hydrogen must be taken into account. More meaningful, however, is to take a closer look at the detailed spectra, which can be found in Figure S4 (Supporting Information) for the C 1s, in Figure S5 (Supporting Information) for the N 1s, and in Figure S6 (Supporting Information) for the O 1s region. Details of the best XPS fits are summarized in Table S1 (Supporting Information). In all samples, the C 1s region is dominated by sp^2^‐hybridized carbon (60.5–67.3 %), whereby the N‐a‐C samples showed a slightly lower sp^2^ contribution. This can be explained by the additional C─N bondings at these samples (a‐N‐C, 17 and 30 nm), which can also be found in the N 1s detail spectra. Here, comparable contributions of mainly pyrrolic (≈50 %), pyridinic (≈26 %) as well as other nitrogen compounds can be found for both N‐a‐C samples. In the case of the 9 and 26 nm a‐C samples, nitrogen was below the limit of detection (LOD). The contribution of oxygen species can also be found because all samples were synthesized at high temperatures and later handled in air.

To gain further information about the carbon structure, Raman microscopy was performed on all samples at ten different positions. Mean spectra are displayed in Figure 1c. The spectra are dominated by a double peak, which is assigned to a contribution of the G band (≈1598 cm^−1^) and of the D band (≈1370 cm^−1^). Both peaks are slightly broader for the N‐a‐C samples, and between these two bands, additional band contributions from C─N vibrations increase the inter‐band region (1400–1500 cm^−1^).^[^ ^34^ ^]^ A deconvolution of the bands in the first order Raman region can be found in Figure S7 (Supporting Information), with details of the best fits in Table S2 (Supporting Information). Here, a fitting with four bands was conducted as suggested for other N‐doped carbon materials.^[^ ^35^ ^]^ The deconvolution shows that both types of samples have very similar results of the band contributions without a particular effect of the sample thickness. In case of the N‐a‐C samples, the D band is shifted by ≈18 cm^−1^ to higher wavenumbers and its full width at half maximum (FWHM) is strongly increased. Furthermore, the contribution of the G band to the overall spectra is significantly decreased at these samples, which indicates an increased disorder, presumably due to the N‐doping.^[^ ^36^ ^]^ Again, in the spectra of the thinner samples (Figure 1c, black and green curves), the contribution of Si─O vibrations from the quartz glass substrate in the low wavenumber region (≈400 cm^−1^) can be found.^[^ ^37^ ^]^ As a consequence, a penetration depth of 25–30 nm of the 532 nm laser can be assumed for these materials. From all the characterization results above, the samples can be treated as partially amorphous, hydrogenated, layered carbon films with nanostructured surface topography. In the case of the acetonitrile precursor, predominantly pyrrolic and pyridinic nitrogen is present in the structure, and both contribute to the defects in the carbon lattice.

**Figure 2:**
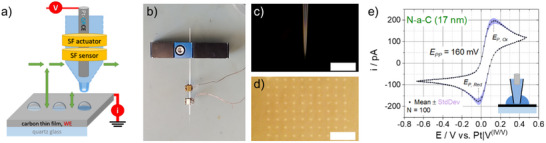
a) Scheme of the SECCM setup, b) probe mounted to the holder with shear force (SF) piezo actuators attached to the capillary, c) optical microscopy image of the capillary tip and d) the probed region of the N‐a‐C (17 nm) sample as well as e) mean values and standard deviation of 100 consecutive CV obtained with a capillary filled with 3 m H_2_SO_4_/25 mm VOSO_4_/12.5 mm V_2_O_5 _solution by hopping mode of SECCM. The scale bar in c) and d) represents 20 µm, and the schematic inset in e) visualizes the drop formation at each spot.

A microelectrochemical method was developed in order to analyze amorphous carbon materials with and without N‐doping toward the vanadium(IV/V) redox reaction. The outstanding advantages of this microelectrochemical technique over electrochemical characterization with macroelectrodes are clearly illustrated below and briefly summarized here. On the one hand, localized investigation provides information about the homogeneity of a material in terms of the chosen electrochemical reaction. On the other hand, multiple sets of parameters can be applied to exactly the same thin‐film sample, allowing the sample to be polarized into potential ranges that are normally avoided because they are assumed to severely damage such materials. Therefore, the SECCM technique was modified with respective parameters, and the principal setup and operation mode are schematically displayed in **Figure** **2**a. An equimolar 50 mm vanadium(IV/V) electrolyte in 3 m sulfuric acid, which reflects real battery electrolyte conditions in terms of viscosity, acidity, and sulfate ion concentration, was filled into a prepared microcapillary equipped with a Pt wire. The Pt wire served as a quasi‐reference counter electrode, which provides a constant reference system by the equimolar vanadium(IV/V) electrolyte [E(Pt|V^(IV/V)^)] as well as the counter reaction in the same compartment. For more details, see the Supporting Information. The respective sample was in contact to the capillary opening. The capillary was equipped with two piezo actuators to employ a height control mode Figure 2b). This type of shear force microscopy‐based method is also known from scanning electrochemical microscopy (SECM) as a height control technique.^[^ ^38^ ^]^ The opening of the capillary was <1 µm (Figure 2c), and the capillary formed 2‐3 µm drops on the surface of the sample after approaching, which remained on the sample after retracting the capillary (Figure 2d). Aspects of the approaching and the resulting “hopping” SECCM mode can be found in Figure S8 (Supporting Information) and the text of the Supporting Information.

This mode was used to analyze all samples by conducting a 10 × 10 point mapping (Figure 2d). At each point, cyclic voltammograms (CV) between ‐0.66 and 0.46 V were performed after the approach. Afterward, the capillary was retracted, and the position of the sample was changed by 7 µm, respectively. A mean CV (the second of three consecutive CVs) of the 17 nm N‐a‐C sample from 100 single measurement points is displayed in Figure 2e, showing high reproducibility of the suggested proceeding. In contrast to other SECCM work,^[^ ^39^ ^]^ sigmoidal shape with dominant diffusion control is not present here. Note that the formed drop exceeds the pure capillary diameter (see schematic inset in Figure 2e) because no hydrophibization of the outside of the capillary was carried out. Consequently, both maximum peak currents, anodic and cathodic, reflect the conversion of vanadium(IV or V) species in the drop entity surrounding the capillary (meniscus).^[^ ^40^ ^]^ After passing the maxima in the CV, the current results from the diffusion of species from the capillary to the sample surface. As the electrolyte contains equimolar amounts of redox‐active species, the CV reflects a highly symmetric reaction, which is not always the case at the positive half‐cell of the VRFB.^[^ ^10^ ^]^ Nevertheless, both peak maxima in Figure 2e are not appearing equidistant to E(Pt|V^(IV/V)^) = 0, which might be a result of different adsorption effects of the platinum and carbon. The half‐cell potential E_0_ of the vanadium(IV/V) reaction in 3 m H_2_SO_4_ was determined as 1.08 V vs. RHE (see Supporting Information). This is in accordance to the standard half‐cell potential (E’_0_) of this reaction that shifts to higher potentials with increased sulfuric acid concentration.^[^ ^41^ ^]^

Furthermore, the potential difference of the peak maxima in the CV (E_PP_) is a measure of kinetic activity and reversibility of the reaction. **Table** **1** shows the peak separation from CV analysis of all samples. The direct calculation of rate constants of the reaction from E_PP_ as shown by the work of Nicholson^[^ ^42^ ^]^ and Lavagnini et al.^[^ ^43^ ^]^ cannot be applied here for two main reasons. On the one hand, there is film porosity, which can strongly influence the CV results.^[^ ^44^ ^]^ Here, a distinction must be made between internal film porosity and surface topography. Internal film porosity is negligible, as XRR measurements only provide results for closed, uniform films fully covering the substrate. However, surface topography makes a significant contribution, as discussed above and shown in Figure S3a (Supporting Information). On the other hand, the experimental cell geometry of the microelectrochemistry is significantly different from that of Nicholson with macroelectrodes. Nevertheless, the peak separation (E_PP_) can be taken as a qualitative measure of the reaction kinetics of the samples. As a reference, another prominent, non‐carbon electrode material was probed by the SECCM method. In case of the indium‐doped tin oxide (ITO) electrode, a shifting of the vanadium(IV) oxidation as well as a drastical shift of the vanadium(V) reduction was observed (Figure S8d, Supporting Information), which confirms the sluggish kinetics of this material. In case of the carbon thin film samples, the present results do not indicate an increased activity of the N‐doped samples, although the surface topography is comparable for all samples (cf. Figure S3, Supporting Information).

**Table 1:** Peak separation (E_PP_) from the CV analysis of all samples, as well as the data of an indium‐doped tin oxide (ITO) electrode from the CV in Figure S8d (Supporting Information).

Sample	*E_PP_*/mV
a‐C (9 nm)	120
a‐C (26 nm)	160
N‐a‐C (17 nm)	158
N‐a‐C (30 nm)	160
Indium‐doped tin oxide	870

**Figure 3:**
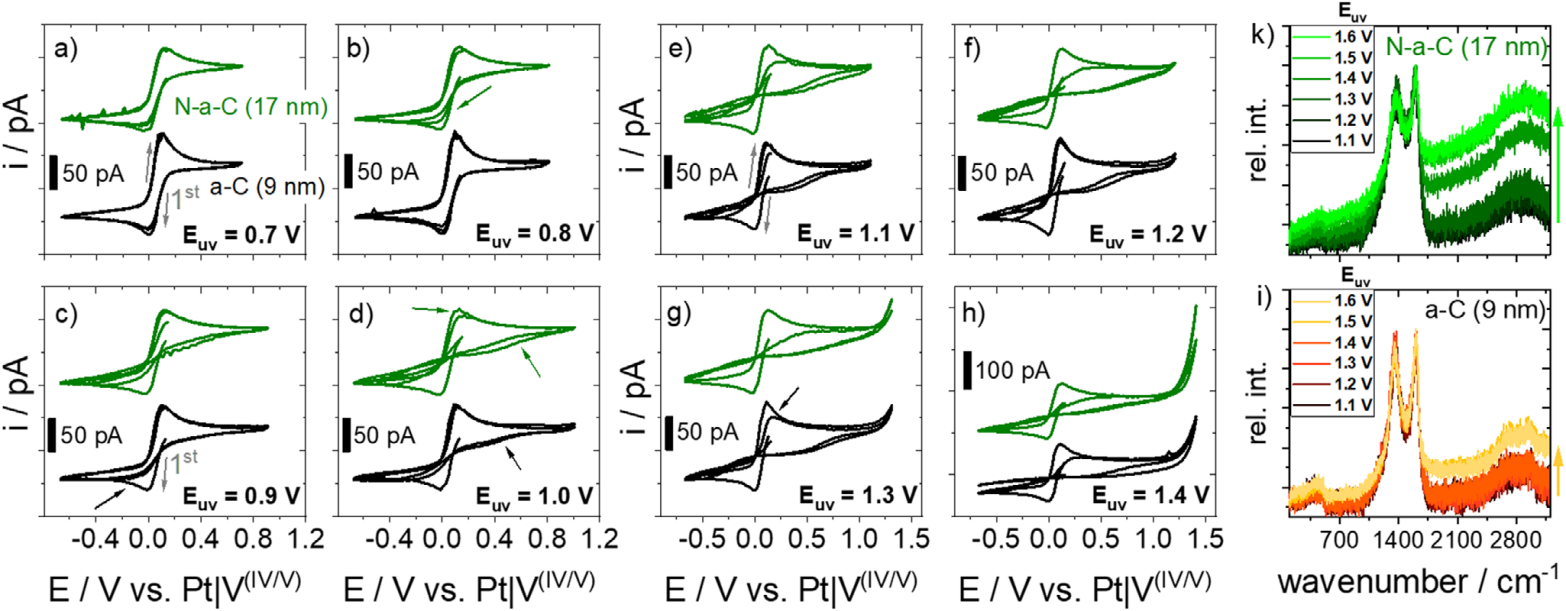
Three consecutive cyclic voltammograms of the 9 nm a‐C and the 17 nm N‐a‐C sample with increased upper vertex potential (E_uv_) of a) 0.7 V, b) 0.8 V, c) 0.9 V and d) 1.0 V for the low overpotential region and e) 1.1 V, f) 1.2 V, g) 1.3 V and h) 1.4 V for the high overpotential region as well as correlated ex situ Raman spectra of the very same SECCM spots of the i) 9 nm a‐C and k) 17 nm N‐a‐C sample after the experiments. The arrows mark important regions or tendencies, which are discussed in the text.

In order to gain deeper insights into the stability against high potentials of such thin film electrodes as well as to study the related influence of the N‐doping, the SECCM method was adjusted in a suitable way, and consecutive CV experiments were performed (**Figure** **3**). Please note that all potentials are given with regard to the Pt|V^(IV/V)^‐QRCE in the following. First, the CV started at 0.15 V into the negative scanning direction in all cases. After passing the lower vertex at ‐0.66 V, it was scanned into the positive direction. Second, the upper vertex potential (E_uv_) of the same CV experiment was adjusted to potentials of the low (Figure 3a–d) and of the high overpotential region (Figure 3e–h) of the vanadium(IV/V) reaction. Note that for each CV measurement, a new position of the sample was approached, which excludes any influence of the previous polarization on the respective SECCM spot. Figure 3 comparatively displays the results of both thinner samples (9 nm a‐C and 17 nm N‐a‐C). In the case of the upper vertex potential (E_uv_) of 0.7 V, a highly symmetric CV can be found for both samples with identical progression of all three consecutive CV. At E_uv_ = 0.8 V, the current of the vanadium(V) reduction is decreased for the 17 nm N‐a‐C sample. The same can be observed for the 9 nm a‐C sample at E_uv_ = 0.9 V (Figure 3c). At E_uv_ = 1.0 V, the current of the vanadium(IV) oxidation is also starting to decrease in the second CV scan in the case of the 17 nm N‐a‐C sample, and a broad additional reduction wave (arrows in Figure 3c) can be found between 0.8 and 0.2 V at both samples in the negative scan direction. In the high overpotential region (Figure 3e–h), this broad peak becomes even more pronounced. At E_uv_ = 1.1 V, the onset of an additional oxidative process can be found, which is the strongest current contribution at 1.4 V for both samples. For the 9 nm a‐C sample, the current of the vanadium(IV) oxidation in the second CV scan is starting to decrease at E_uv_ = 1.3 V, which is significantly higher (300 mV) than in the case of the N‐doped sample. Additionally, the experiments were also conducted for E_uv_ = 1.5 and 1.6 V. At these potentials, the SECCM drop morphology changes drastically, and leakage as well as drop spreading can be observed after reaching the E_uv_ for the first time. As a result, the CV lacks reproducibility and is left out here. Again, it should be stressed that the first CV in a negative potential direction acts as a sample reference to confirm the homogeneity of the probed positions and to exclude any position influence to the discussed results.

**Figure 4:**
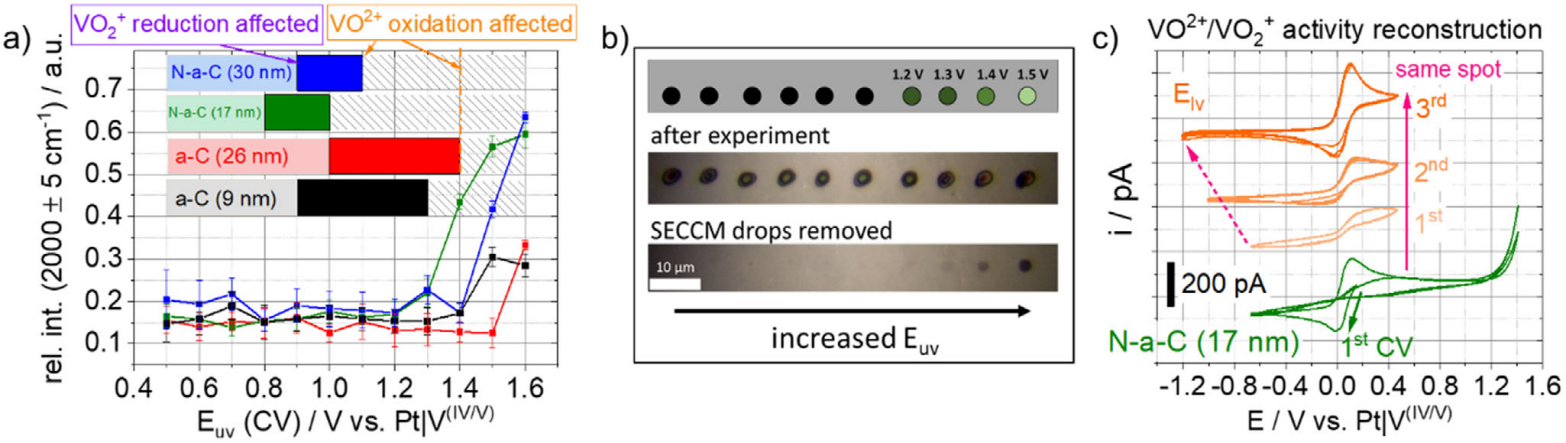
a) Evaluation of CV and Raman microscopy from SECCM experiments in Figure 3 and Figure S9 (Supporting Information), b) scheme of the consecutive SECCM spots of the 17 nm a‐N‐C sample and microscopy images before and after cleaning and drying of the sample and c) consecutive CVs of 17 nm N‐a‐C sample with E_uv_ = 1.4 V (green) and with decreased lower vertex (E_lv_ = ‐0.5 V, ‐1.0 V and ‐1.2 V) of the very same spot (orange).

To get a deeper understanding of the ongoing processes and structural changes, which may happen in particular at high overpotentials (E_uv_: 1.2–1.4 V), ex situ Raman microscopy was performed afterward at the very same spots of probing. Our instrument would also have allowed for in situ characterization, but recent results showed that Raman laser probing in combination with a high electrochemical potential could drastically influence the Raman results.^[^ ^45^ ^]^ From the Raman results in Figure 3k,i, changes of band position (D, G) and intensity ratio were not observed at both samples, indicating that the basic carbon structure remains unchanged. However, a contribution of fluorescence, affecting the Raman spectrum baseline, can be found for the 17 nm N‐a‐C sample starting at 1.3 V and at 1.5 V for the 9 nm a‐C sample. With some slight deviation in the respective potentials, these results and observations from CV and consecutive Raman probing are comparable to those of the thicker samples (26 nm a‐C and 30 nm N‐a‐C), and a summarizing overview can be found in **Figure** **4**a. The respective results of both thicker samples can be found in Figure S9 (Supporting Information).

In Figure 4a the upper vertex potentials, at which the vanadium(V) reduction as well as the vanadium(IV) oxidation is affected, are marked with colored bars, where the lighter colors represent the region without effects on the redox features and the darker colors the beginning of the VO_2_^+^ reduction and consecutive VO^2+^ oxidation peak potential shift and current decrease. In the potential region with striped bars, both reactions are affected. In general, the reduction is affected when potentials from 0.8 to 1.0 V are reached during the CV. Here, the appearance of VO_x_ surface intermediates formed at high potentials might explain the hindered reduction. Another more suitable explanation can be found by considering the appearance of an anion insertion process at the samples. In the case of graphite, this competitive process connected with the disappearance of the vanadium(V) reduction has already been reported in early work.^[^ ^15^ ^]^ In all cases, the hindered vanadium(V) reduction is accompanied by an additional reduction wave (see Figure 3d). Furthermore, this wave is drastically more pronounced for the thinner samples (9 and 17 nm, Figure S10a,c, Supporting Information) than for the thicker ones (26 and 30 nm Figure S10b,d, Supporting Information) which allows for the conclusion of a thickness‐depending phenomenon. A deposition process (VO_x_ formation at the electrode surface) is expected to appear independently of the electrode layer thickness when conductivity is not limited. It is therefore obvious that other processes, such as (anion) insertion reactions, well known as intercalation in the case of graphite, must also be taken into account for amorphous carbon materials at these high potentials. Insertion reactions are well‐understood processes and can be highly reversible. Consequently, the counter reaction, which is known as ion extraction, appears when the CV is scanned back into the negative direction and is competitive to the vanadium(IV/V) redox processes. From this point of view, the additional reductive wave (Figure S10, Supporting Information and marked with arrows in Figure 3d) in the backward scan can be interpreted as a change from diffusion‐controlled vanadium(IV) oxidation to a thermodynamically more favored reaction, which in this case might be the sulfate ion extraction. However, the ion extraction seems to depend on the composition of the carbon substrate here (Figure 4a). In the case of the N‐doped samples (N‐a‐C), the time frame of the scan in the negative potential direction seems not to be sufficient to finish the process, and, as a result, the oxidation of vanadium(IV) in the consecutive forward scan of the CV is earlier affected at a lower potential than in the case of the a‐C samples. The difference of 300 to 400 mV is a strong hint that this insertion compound is much better stabilized in the N‐doped carbon than in the undoped carbon samples.

Furthermore, ex situ Raman microscopy of the SECCM spots results in strong fluorescence in the spectrum of the N‐a‐C samples, which cannot be found for the a‐C samples in the studied potential region. This leads to the assumption that both the early appearance of the additional reduction wave and the Raman fluorescence of the substrate are connected to each other. The fluorescence in the Raman spectra is accompanied by a strong current increase in CV beginning at potentials >1.1 V (Figure 3f–h). However, this increase in current cannot necessarily be attributed to any type of gas evolution reactions (O_2_ or CO_2_) because the SECCM probe was used right after for the next sample probing. Bubble formation could easily block the capillary opening, preventing any current flow afterward, which was not the case in any of the experiments. This leads to the hypothesis that the processes appearing at the samples at high potentials are an ongoing insertion process affecting additional carbon layers with possible enlargement of the layer distance of the sp^2^‐rich carbon thin films. Scanning electron microscopy images showed a layered structure of the samples (Figure S1c,d, Supporting Information), which can act as a host structure for the anion insertion reaction. Furthermore, this finding is supported by inspecting the SECCM sample spots by optical microscopy. Individual spots, presumably those polarized to potentials >1.1 V, showed changes in their optical appearance. In Figure 4b, images of the spots of the prior SECCM probing of the 17 nm N‐a‐C sample are shown before (drops are present) and after cleaning with water and drying. At E_uv_ = 1.2 V a greyish shadow is already present, and the spots become darker with increased E_uv_. A dark black spot can be observed at 1.5 V. Consequently, a structural change of the thin film has to be present, although the Raman features (D and G band) of the carbon remain unchanged if the fluorescence contribution is neglected (Figure 3i,k). The optical changes at the spots of the 17 nm N‐a‐C sample in dependence of E_uv_ allow for the conclusion that starting at 1.2 V, the insertion process affects more and more layers, and an irreversible change of the interlayer distance may appear. This process is also known as surface swelling,^[^ ^46^ ^]^ which can also affect the optical properties.

To better understand this process, it is necessary to classify the significance of the fluorescence of carbon materials. Fluorescence, as observed in Figure 3k and i as well as in Figure S9k and i (Supporting Information), is known but only partially understood when referring to “carbon (quantum)dots” which are nanosized (1–10 nm) carbon monolayered structures synthesized under harsh conditions by ultrasonication, electrochemical oxidation and/or exfoliation, arc discharge or laser ablation from various carbon sources.^[^ ^47^ ^]^ All these processes must be connected to an ion insertion reaction that separates the monolayers. However, insertion reactions can be far better studied at highly‐ordered graphite structures where the graphite intercalation compound (GIC) can be synthesized by similar processes. Here, fluorescence at high wavenumbers is described in a few literature for a hindered stage‐1 sulfate‐GIC where the origin of the hindrance is deduced to be unclear.^[^ ^48^ ^]^ Dimiev et al. postulated a pseudoamorphous state with strong fluorescence contribution in the Raman spectra, which appears during the stage‐1 to stage‐2 GIC transformation process.^[^ ^49^ ^]^ As a consequence, fluorescence can be interpreted as an indicator of the proceeded ion insertion at amorphous carbon materials here. However, it cannot be referred to a defined stage as in the GIC but it might be comparable to the stoichiometry of a stage‐1/2 GIC. It is known from the literature that the initial beginning of the sulfate insertion (onset) can appear more than 1.0 V earlier,^[^ ^50^ ^]^ which is 0.2 V or lower in our case. As a result, the beginning insertion of sulfate ions can already appear close or in parallel to the vanadium(IV) oxidation.

To prove the high reversibility of the ion insertion‐extraction processes, selected sample spots were polarized to lower potentials that favor the reverse reaction. For the N‐a‐C (17 nm) sample, cyclic voltammograms to decreased vertex potentials (orange curves) are shown in Figure 4c for the very same spot that was prior polarized to 1.4 V by CV (green curve). Deactivation of the vanadium(IV/V) redox reaction is observed as before, but apparently its prior redox activity can be fully reconstructed by consecutive CVs with a lower vertex potential (E_lv_ = −1.2 V). This observation confirms the reversibility of the previously mentioned process. Moreover, the material conductivity at the harmed spot seems to be unchanged as the vanadium(IV/V) peak separation (E_PP_) is not affected. This finding again stresses that irreversible processes like carbon corrosion as well as the oxidation of the carbon‐carbon structure or the π‐system are not predominant here.

**Figure 5:**
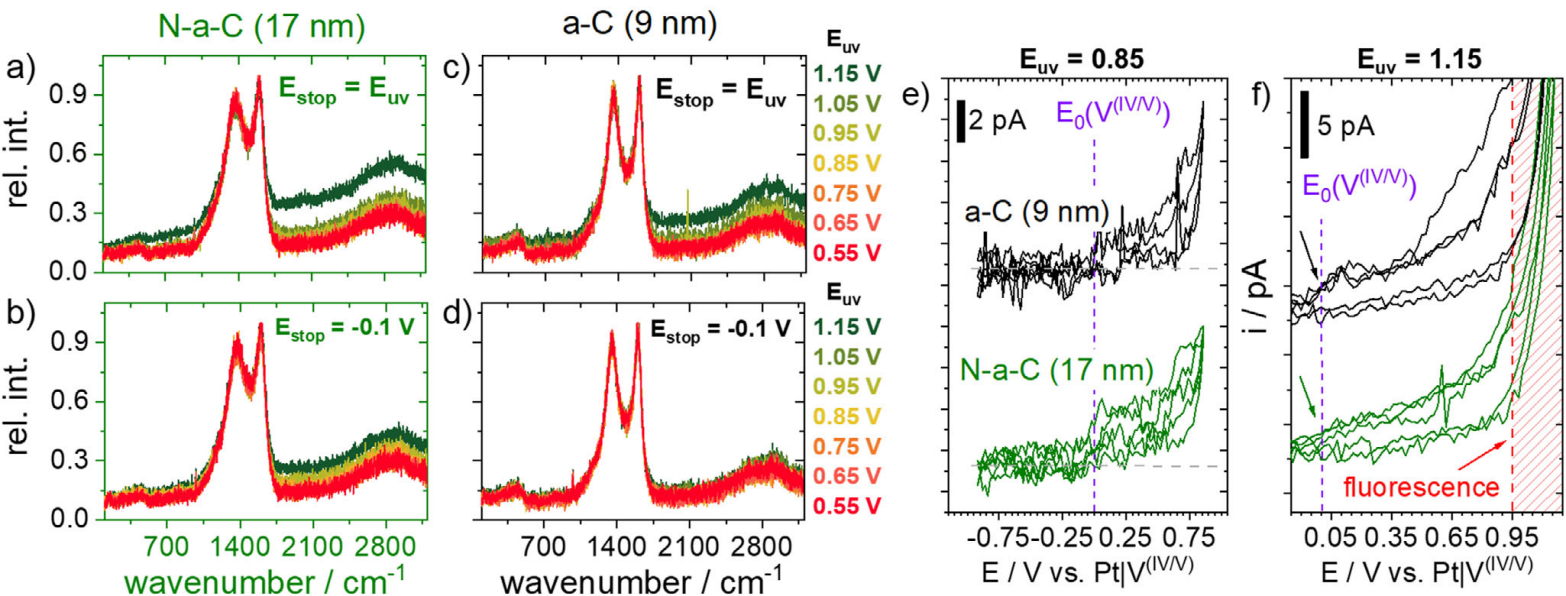
Raman spectra of the spots after SECCM probing with a capillary filled only with 3 m H_2_SO_4_ with a related experimental processing as in Figure 3 for a) and b) the N‐a‐C (17 nm) as well as c) and d) the a‐C (9 nm) sample. The CV was stopped once at the upper vertex potential (E_uv_) (a and c) and once at ‐0.1 V (b and d). Selected corresponding cyclic voltammograms of the 9 nm a‐C and the 17 nm N‐a‐C sample with the upper vertex potential (E_uv_) of e) 0.85 V and f) 1.15 V, with E_stop_ = ‐0.1 V. The dashed grey lines in e) represent I = 0 A, and the arrows in f) indicate the beginning double layer increase at carbon thin films.

To gain more profound data about the initial appearance of the ion insertion reaction, adapted experiments were also conducted with the SECCM probe containing solely 3 m H_2_SO_4_ electrolyte. Despite the absence of V‐containing components, it should be noted that all of the following potentials have been converted to Pt|V^(IV/V)^‐QRCE for better comparability (see Supporting Information). The upper vertex potential (E_uv_) was increased from 0.55 to 1.15 V for the 17 nm N‐a‐C (**Figure** **5**a,b) and the 9 nm a‐C (Figure 5c,d) sample in two different ways. First, the initial beginning of fluorescence of both samples was determined by stopping the CV probing at the upper vertex potential (E_uv_). In contrast to the procedure before, the formed insertion is retained and not dissolved by the last backward scan. Comparing the results with E_stop_ = E_uv_, an identical beginning of the fluorescence can be found at 0.95 V (light green spectra in Figure 5a,c evolve from the previously uniform spectra). Fluorescence increases in both groups of spectra with increasing E_uv_. Comparing the spectra at 1.15 V, the effect is again stronger for the 17 nm N‐a‐C sample. This experiment clearly confirms the dominant involvement of sulfate anions to the prior observations. Second, the reversibility is analyzed by stopping the CV after a full scan back to lower potentials (E_stop_ = −0.1 V, Figure 5b,d). Here, the fluorescence fully disappeared at the 9 nm a‐C sample but only partially at the 17 nm N‐a‐C sample. This is in accordance to the prior results and points again to a more stable insertion compound of the N‐a‐C sample. Obviously, the sulfate extraction cannot be completed in the chosen potential or timeframe. This discrepancy may explain why the consecutive oxidation of vanadium(IV) is far more affected at the N‐doped samples (cf. Figure 4a). Again, it should be noted that according to the work of Dimiev et al. ^[^ ^49^ ^]^ fluorescence appears on a pseudoamorphous transition stage between stage−1 and −2 in GIC. Consequently, the onset of fluorescence marks the end of stoichiometric incorporation into a certain number of (surface‐near) layers, since fluorescence is much stronger than Raman scattering. Increasing fluorescence contribution might be interpreted as an increased number of affected carbon layers reaching such a stage.

**Figure 6:**
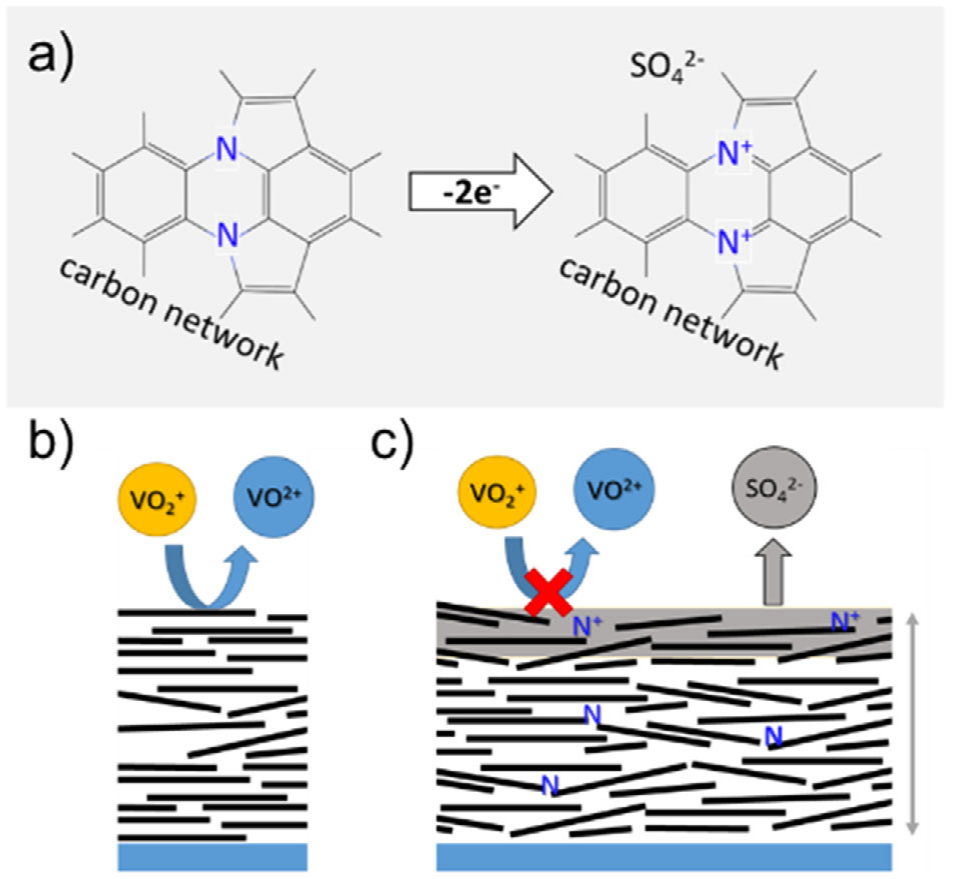
a) A simplified scheme of the formation of electron‐deficient carbon‐nitrogen structures (pyrrolium‐like here) within the carbon network with electrostatic compensation of sulfate anions, b) schematic representation of the competing processes appearing at a‐C and c) predominantly at N‐a‐C thin film samples.

To further clarify, where the beginning of the insertion process can be found, selected CVs in only sulfuric acid for E_uv_ = 0.85 and 1.15 V are displayed in Figure 5e,f, respectively. However, the following observations can also be found in all other CVs. Figure 5e,f shows CVs where the current predominantly is a result of double layer formation from the electrode spot in the sulfuric acid electrolyte. It should be mentioned that the very low currents, primarily through mainly capacitive contributions, lead to relatively noisy CVs. In Figure 5e, the double layer increases when the CV is scanned to a potential >0.05 V. In the consecutive CV scan, this double layer increase vanishes again at potentials <0.05 V, which again points to a reversible process. In our opinion, the change in the double layer contribution reflects a stronger interaction of sulfate ions with the samples. The early begin of the first layer intercalation of sulfate in highly‐oriented pyrolytic graphite (HOPG) has already been confirmed by droplet‐electrowetting technique.^[^ ^51^ ^]^ Thus, it might also be the situation here that insertion into surface layers starts rather early but becomes a predominant bulk phenomenon at much more positive potentials. From this perspective, the observed changes in the CV at potentials <0.05 V can also be interpreted as pseudo‐capacitive. In Figure 5f, a strong current increase is observed at 0.95 V, accompanied by fluorescence as stated before. This is slightly earlier compared to the results in Figure 3f in the presence of vanadium ions. The ongoing insertion of sulfate ions forms a carbon insertion compound, which is much more stable at the N‐doped samples as indicated by the Raman results (Figure 5a–d). In **Figure** **6**a, a possible explanation of the increased stability of this carbon insertion compound is given. In contrast to carbon, pyrrolic and pyridinic nitrogen, as proved by XPS analysis (see Figure S5, Supporting Information), can compensate for missing electrons of the electrochemical oxidation by the formation of electron‐deficient carbon‐nitrogen moieties structures (pyrrolium‐ and pyridinium‐like), which can be electrostatically compensated by the presence of sulfate anions. These structures are far more stable than (C)_n_^+^, which is formed in the carbon lattice at high potentials.

Summarizing all the results of applying high overpotentials to these types of carbon samples, the findings of competing reactions, as well as the influence of the N‐doping of hydrogenated carbon electrodes, can be discussed comprehensively. A schematic illustration is given in Figure 6b,c. Initially, all samples showed high reversibility toward the vanadium(IV/V) reaction without any particular contribution of the pyrrolic/pyridinic nitrogen moieties. At potentials higher than 0.8 V, the vanadium(V) reduction is affected by the presumable formation of a sulfate insertion compound, which cannot be fully reversed, accompanied by an apparent broad reduction wave in the backward scan of the CV. At potentials lower than 0.8 V this reaction may also appear, but the effects are completely reversed in the backward CV scan. Therefore, corresponding measurements in solely sulfuric acid electrolyte indicate that the initial sulfate insertion may already be present at 0.0 V, which is the equilibrium potential of the vanadium(IV/V) reaction here (Figure 5e,f). In contrast to the a‐C samples, the anion extraction is not fully completed at the N‐a‐C samples within the chosen potential region and time frame of the backward CV scan, which leads to a hindrance of the consecutive vanadium(IV) oxidation in the following CV. At the N‐a‐C samples, the deactivation of the vanadium(IV) oxidation appears 300–400 mV earlier than in the case of the undoped a‐C samples, regardless of the layer thickness (Figure 4a). This finding allows for the conclusion that the insertion compound is stabilized by the presence of nitrogen‐containing structural features, which slows down the anion extraction process by the formation of an electron‐deficient carbon‐nitrogen structure (Figure 6a). A further increase of the potential to the high overpotential region leads to an additional oxidative current beginning at 1.1 V. In the case of solely sulfuric acid electrolyte, the beginning already appears at 0.95 V, which might be a result of the missing competitive vanadium(IV) oxidation. This beginning corresponds to the appearance of fluorescence in the Raman spectra at both types of sample, which here can be assigned to a changed stoichiometry of the insertion compound at the amorphous carbon thin films. Due to the amorphous nature of the samples, the exact stoichiometry, which is known as “stage” in the case of graphite, cannot be determined, but fluorescence can be found in the case of stages comparable to GIC stage 1‐2 transition in other work.^[^ ^49^ ^]^ Consequently, a further increase of the potential might also separate individual layers, as it is known from the exfoliation of graphitic carbon.^[^ ^52^ ^]^ Again, this reaction is much more pronounced, and the formed insertion compound is more stable at the N‐a‐C samples, but can be fully reversed by polarizing the sample to lower potentials for a certain time. Consecutive Raman spectra show that the basic carbon structure of these thin film electrodes remains unaffected even after polarizing to 1.4 V in the CV. The insertion‐extraction process seems to be slow, as expected for diffusion processes, and can be affected within the time frame of the CV probing. Finally, all samples showed initially high stability against oxidative potentials, and the carbon corrosion or oxidation processes do not appear as the predominant process in the analyzed potential region and time frame for these types of samples.

## 3 Conclusion

In this work, thin film hydrogenated carbon substrates with and without N‐doping were analyzed toward the vanadium(IV/V) reaction by SECCM technique using an electrolyte, which mimics VRFB conditions. Evaluation of the peak separation as a kinetic parameter could not confirm a rate constant enhancement by the N‐doping. The high overpotential region of the reaction was probed by increasing the upper vertex potential of the presented CV method. The formation of a sulfate insertion compound appears in competition to the redox reaction beginning at 1.1 V vs. RHE, which is close to the equilibrium potential of the vanadium(IV/V) reaction. At potentials above 1.9 V vs. RHE, its formation affects the vanadium (V) reduction, and the ongoing insertion compound formation becomes electrochemically dominant at 2.3 V vs. RHE. The formation is favored by N‐doping of the carbon, which might be explained by the electrostatic compensation of an electron‐deficient carbon‐nitrogen structure within the material. As expected, the insertion reaction depends on the sample thickness and is fully reversible; however, the stability of the N‐doped carbon‐sulfate compound prolongs the extraction process. In this work, there is no evidence of VO_x_ adsorbate formation, which may require the use of other powerful surface spectroscopic techniques. However, it cannot be excluded that the insertion in some way is additionally influenced by the presence of the vanadium ions. The features of the vanadium(IV/V) reaction as well as the Raman spectrum of the carbon thin films, remain unaffected even after applying 2.5 V vs. RHE, when the sulfate insertion reaction is reversed by suitable low potentials. This remarkable value reflects the impressive stability of this type of carbon and indicates a minor role of irreversible carbon corrosion processes. Finally, further experiments are necessary to clarify the extent to which the observed insertion‐extraction process contributes to other degradation and/or carbon corrosion phenomena, particularly with regard to long‐term stability during potential or charge‐discharge cycling.

## 4 Experimental Section

### Synthesis

An ambient pressure‐chemical vapor deposition setup (AP‐CVD) was used to synthesize the carbon thin films. Quartz glass substrates (2 × 2 cm^2^, PGO GmbH) were cleaned consecutively with hydrochloric acid, acetone, and water in a beaker in an ultrasonic bath (Bandelin). The quartz substrate was then placed in a quartz glass tube (35 mm in diameter) in an oven (Carbolite) connected to calibrated mass flow controllers (MFC, Bronkhorst). The temperature was increased to 1085 °C with 10 K min^−1^ under constant Ar gas flow (20 l h^−1^). After reaching 1085 °C the gas flow and composition was changed to 5 l h^−1^ of a mixture of argon and hydrogen (Ar:H_2_, 97:3). This mixture was used to transport a saturated gas volume over a liquid of acetonitrile (HPLC grade, Fisher Scientific) in case of the N‐doped, amorphous carbon (N‐a‐C) samples or cyclohexane (>99.5%, Carl Roth) in case of the amorphous carbon (a‐C) samples to the oven over a time period of 10 to 60 s. From greyish, semitransparent to mirror‐like appearing samples (see Figure S1, Supporting Information) were obtained in dependence of the sample thickness.

### Morphological Characterization

Raman spectra were obtained using a commercial InVia spectrometer setup (Renishaw) with Leica DMI 3000 microscope, 532 nm laser, 1800 l mm^−1^ grating, Rayleigh filter, and CCD camera. Before use, the instrument was calibrated using a polycrystalline silicon reference, and the signal was adjusted to 520.4 cm^−1^. Laser intensities were adjusted in a way that the sample spots were not harmed by the radiation.

A commercial four‐point‐probe station (Ossila, UK) with a gold contact probe head was used to determine the sheet resistance of the thin film samples. The current range was adjusted for each sample separately, and the data were evaluated by the Ossila software. Each sample was probed at different positions several times.

A commercial high‐resolution diffractometer (Bruker D8 Discover) with monochromatic Cu Kα1 radiation (λ = 1.54056 Å) was used to analyze the small angle region of the thin film samples from 0.3° to 4° 2θ with a step size of 0.01°. Film thickness was calculated by evaluating the Kiessing oscillation using Bragg`s equation. Furthermore, XRD measurements were performed for individual samples in the Bragg–Brentano geometry, changing 2θ from 5° to 50° with a step size of 0.02°.

Infrared (IR) spectra were recorded using a Vertex 70v FT‐IR spectrometer (Bruker) with a SiC‐based MIR source, a KBr beam splitter, and an LN‐MCT D313 detector. In transmission mode, spectra from 128 scans in the range of 4000–400 cm^−1^ with a resolution of 4 cm^−1^ were obtained (aperture: 2 mm, mirror velocity: 5 kHz). Background correction was performed by measuring the spectrometer without a sample, using the same parameters.

The surface composition of selected thin film samples was analyzed by X‐ray photoelectron spectroscopy (Omicron UHV system with a hemispherical EA125X electron energy analyzer with a 5 channeltron detector and DAR 400 X‐Ray source with Al Kα radiation). The pass energy was 100 eV for the survey scan and 30 eV for spectra of the C 1s, N 1s, and O 1s regions. Casa XPS software was used for peak deconvolution of the detail spectra.

Scanning electron microscopy (SEM) images were obtained using a Zeiss Gemini 500 field emission scanning electron microscope (FE‐SEM) with an acceleration voltage of 1–2 kV.

### Scanning Electrochemical Cell Microscopy (SECCM)

A modified SECM/SECCM setup (Sensolytics GmbH) mounted on top of an inverted microscope^[^ ^27^ ^]^ was used for the microelectrochemical probing. SECCM probes were fabricated from borosilicate glass capillaries with filament (BF100‐58‐15, Science Products GmbH), using a Sutter 2000 laser puller with the following parameters: HEAT = 275, FIL = 4, VEL = 50, DEL = 225, PULL = 150.^[^ ^28^ ^]^ Fabricated capillaries were filled with electrolyte by a syringe and a small syringe needle (MF34G, World Precision Instruments). The electrolyte consisted of 0.025 m vanadium(IV) oxide sulfate hydrate (VOSO_4_·xH_2_O, 97%, Sigma–Aldrich) and 0.0125 m vanadium(V) oxide (V_2_O_5_, >98%, Sigma–Aldrich) dissolved in 3 m sulfuric acid (98 wt.%, Carl Roth GmbH). Due to the slow dissolution of the vanadium(V) oxide, the mixture was treated in a temperature‐controlled ultrasonification bath (Sonocool, Bandelin GmbH) until a clear, light greenish solution was obtained. The vanadium(IV) content was confirmed by potentiometric titration (877 Titrino plus, Metrohm) using commercial 0.1 m cerium(IV) sulfate titration solution (Carl Roth GmbH).

The SECCM capillary probes were equipped with two shear force (SF) piezo actuators (Sensolytics GmbH), which were used for a controlled approach to the sample surface and to perform a “hopping” mode SECCM experiment. Furthermore, a platinum wire (99.999%, 250 µm diameter, Goodfellow) was inserted without any further pretreatment. The carbon thin film samples were contacted by a copper adhesive tape ≈5 mm from each side of the square, to obtain homogeneous film conductivity. After approaching the SECCM capillary to the sample, cyclic voltammetry (CV) was conducted in a two‐electrode configuration where the sample acts as the working and the platinum wire in the capillary as a quasi‐reference counter electrode (Pt‐QRCE). CVs were obtained with a scan rate of 200 mV s^−1^ and a step size of 20 mV. Upper (E_uv_) and lower vertex potential (E_lv_) of the CVs were adjusted with respect to the experimental processing and are discussed in detail in the results section. Besides the carbon thin films, an indium‐doped tin oxide (ITO) electrode (≈100 nm on quartz glass, pgo GmbH) was used as reference material.

### Statistical Analysis

In the case of the Raman microscopy probing as well as the SECCM mapping experiments of the thin film samples, multiple data sets were acquired. The mean value and standard deviation were calculated and presented in the respective figure together with the sample size (N). All Raman spectra were normalized to the maximum peak intensity before calculation. The CV data were used without any further pre‐processing.

## Conflict of Interest

The authors declare no conflict of interest.

## Supporting information



Supporting Information

## Data Availability

The data that support the findings of this study are available from the corresponding author upon reasonable request.

## References

[1] K. J. Kim , M.‐S. Park , Y.‐J. Kim , J. H. Kim , S. X. Dou , M. Skyllas‐Kazacos , J. Mater. Chem. A 2015, 3, 16913.

[2] L. Ye , S. Qi , T. Cheng , Y. Jiang , Z. Feng , M. Wang , Y. Liu , L. Dai , L. Wang , Z. He , ACS Nano 2024, 18, 18852. 38993077 10.1021/acsnano.4c06675

[3] M. Shoaib , P. Vallayil , N. Jaiswal , P. I. Vaigunda Suba , S. Sankararaman , K. Ramanujam , V. Thangadurai , Adv. Energy Mater. 2024, 14, 2400721.

[4] N. Remmler , M. Bron , ChemElectroChem 2024, 11, 202400127.

[5] T. Cheng , S. Qi , Y. Jiang , L. Wang , Q. Zhu , J. Zhu , L. Dai , Z. He , Small 2024, 20, 2400496. 10.1002/smll.202400496 38949033

[6] J. Wu , R. Nie , L. Yu , Y. Nie , Y. Zhao , L. Liu , J. Xi , Small 2024, 20, 2405643. 10.1002/smll.202405643 39308314

[7] N. Roznyatovskaya , J. Noack , K. Pinkwart , J. Tübke , Curr. Opin. Electrochem. 2020, 19, 42.

[8] Y. Nie , R. Nie , H. Lin , J. Wu , L. Yu , L. Liu , J. Xi , Angew. Chem., Int. Ed. 2025, 64, 202420794. 10.1002/anie.202420794 39688466

[9] N. Kausar , R. Howe , M. Skyllas‐Kazacos , J. Appl. Electrochem. 2001, 31, 1327.

[10] M. Gattrell , J. Qian , C. Stewart , P. Graham , B. MacDougall , Electrochim. Acta 2005, 51, 395.

[11] W. Wang , X. Fan , J. Liu , C. Yan , C. Zeng , Phys. Chem. Chem. Phys. 2014, 16, 19848. 25135306 10.1039/c4cp02416h

[12] W. Wang , X. Fan , Y. Qin , J. Liu , C. Yan , C. Zeng , Electrochim. Acta 2018, 283, 1313.

[13] M. Rakib , G. Durand , Hydrometallurgy 1996, 43, 355.

[14] S. Zhong , M. Skyllas‐Kazacos , J. Power Sources 1992, 39, 1.

[15] H. Kaneko , K. Nozaki , Y. Wada , T. Aoki , A. Negishi , M. Kamimoto , Electrochim. Acta 1991, 36, 1191.

[16] M. Noel , R. Santhanam , J. Power Sources 1998, 72, 53.

[17] D. C. Alsmeyer , R. L. McCreery , Anal. Chem. 2002, 64, 1528.

[18] W. H. Danial , N. A. Norhisham , A. F. Ahmad Noorden , Z. A. Majid , K. Matsumura , A. Iqbal , Carbon Lett. 2021, 31, 371.

[19] H. Radinger , ChemPhysChem 2021, 22, 2498. 34643328 10.1002/cphc.202100623 PMC9297873

[20] L. Wu , Y. Shen , L. Yu , J. Xi , X. Qiu , Nano Energy 2016, 28, 19.

[21] M. A. Costa de Oliveira , C. Schröder , M. Brunet Cabré , H. Nolan , A. Forner‐Cuenca , T. S. Perova , K. McKelvey , P. E. Colavita , Electrochim. Acta 2024, 475, 143640.

[22] Z. Lai , D. Li , S. Cai , M. Liu , F. Huang , G. Zhang , X. Wu , Y. Jin , Anal. Chem. 2023, 95, 357. 36625128 10.1021/acs.analchem.2c04551

[23] R. Jin , H. Lu , L. Cheng , J. Zhuang , D. Jiang , H.‐Y. Chen , Fundam. Res. 2022, 2, 193. 38933173 10.1016/j.fmre.2021.08.001 PMC11197576

[24] M. B. Cabré , C. Schröder , F. Pota , M. A. C. de Oliveira , H. Nolan , L. Henderson , L. Brazel , D. Spurling , V. Nicolosi , P. Martinuz , M. Longhi , F. Amargianou , P. Bärmann , T. Petit , K. McKelvey , P. E. Colavita , Small Methods 2024, 9, 2400639. 39155797 10.1002/smtd.202400639 PMC11740950

[25] C. Gao , Y. Li , J. Zhao , W. Sun , S. Guang , Q. Chen , Anal. Chem. 2023, 95, 10565. 37392190 10.1021/acs.analchem.3c00255

[26] M. A. C. de Oliveira , M. Brunet Cabré , C. Schröder , H. Nolan , F. Pota , J. A. Behan , F. Barrière , K. McKelvey , P. E. Colavita , Small 2024, 21, 2405220. 39548927 10.1002/smll.202405220 PMC11753488

[27] M. Steimecke , G. Seiffarth , M. Bron , Anal. Chem. 2017, 89, 10679. 28933151 10.1021/acs.analchem.7b01060

[28] Y. Shan , N. Panday , Y. Myoung , M. Twomey , X. Wang , W. Li , E. Celik , V. Moy , H. Wang , J. H. Moon , J. He , Macromol. Biosci. 2016, 16, 599. 26757346 10.1002/mabi.201500320

[29] J. Sun , Y. Chen , M. K.r. Priydarshi , Z. Chen , A. Bachmatiuk , Z. Zou , Z. Chen , X. Song , Y. Gao , M. H. Rümmeli , Y. Zhang , Z. Liu , Nano Lett. 2015, 15, 5846. 26305883 10.1021/acs.nanolett.5b01936

[30] C. Lenardi , M. Baker , V. Briois , L. Nobili , P. Piseri , W. Gissler , Diam. Relat. Mater. 1999, 8, 595.

[31] T. Cui , R. Lv , Z.‐H. Huang , H. Zhu , J. Zhang , Z. Li , Y. Jia , F. Kang , K. Wang , D. Wu , Carbon 2011, 49, 5022.

[32] L. Valdes , Proc. IRE 1954, 42, 420.

[33] T. Poche , R. Chowdhury , S. Jang , Mater. Chem. Phys. 2024, 326, 129787.

[34] M. Ayiania , E. Weiss‐Hortala , M. Smith , J.‐S. McEwen , M. Garcia‐Perez , Carbon 2020, 167, 559.

[35] T. Sharifi , F. Nitze , H. R. Barzegar , C.‐W. Tai , M. Mazurkiewicz , A. Malolepszy , L. Stobinski , T. Wågberg , Carbon 2012, 50, 3535.

[36] A. C. Ferrari , S. E. Rodil , J. Robertson , Phys. Rev. B 2003, 67, 155306.

[37] R. H. Stolen , E. P. Ippen , A. R. Tynes , Appl. Phys. Lett. 1972, 20, 62.

[38] B. Ballesteros Katemann , A. Schulte , W. Schuhmann , Chem.‐Eur. J. 2003, 9, 2025. 12740850 10.1002/chem.200204267

[39] M. E. Snowden , A. G. Güell , S. C. S. Lai , K. McKelvey , N. Ebejer , M. A. O'Connell , A. W. Colburn , P. R. Unwin , Anal. Chem. 2012, 84, 2483. 22279955 10.1021/ac203195h

[40] G. Arruda de Oliveira , M. Kim , C. S. Santos , N. Limani , T. D. Chung , E. B. Tetteh , W. Schuhmann , Chem. Sci. 2024, 15, 16331. 39309094 10.1039/d4sc04407j PMC11409436

[41] G. F. Smith , W. M. Banick , Talanta 1959, 2, 348.

[42] R. S. Nicholson , Anal. Chem. 1965, 37, 1351.

[43] I. Lavagnini , R. Antiochia , F. Magno , Electroanalysis 2004, 16, 505.

[44] S.‐J. Kinkelin , F. Röder , K. Vogel , M. Steimecke , M. Bron , Electrochim. Acta 2024, 488, 144183.

[45] S.‐J. Kinkelin , M. Steimecke , M. Bron , Electrochim. Acta 2024, 505, 144991.

[46] M. S. Jagadeesh , A. Calloni , I. Denti , C. Goletti , F. Ciccacci , L. Duò , G. Bussetti , Surf. Sci. 2019, 681, 111.

[47] M. Alafeef , I. Srivastava , T. Aditya , D. Pan , Small 2024, 20, 2303937. 10.1002/smll.202303937 37715112

[48] S. Seiler , C. E. Halbig , F. Grote , P. Rietsch , F. Börrnert , U. Kaiser , B. Meyer , S. Eigler , Nat. Commun. 2018, 9, 836. 29483555 10.1038/s41467-018-03211-1 PMC5826935

[49] A. M. Dimiev , G. Ceriotti , N. Behabtu , D. Zakhidov , M. Pasquali , R. Saito , J. M. Tour , ACS Nano 2013, 7, 2773. 23438444 10.1021/nn400207e

[50] F. Kang , T.‐Y. Zhang , Y. Leng , J. Phys. Chem. Solids 1996, 57, 883.

[51] G. Zhang , M. Walker , P. R. Unwin , Langmuir 2016, 32, 7476. 27406680 10.1021/acs.langmuir.6b01506

[52] H. Lee , J. I. Choi , J. Park , S. S. Jang , S. W. Lee , Carbon 2020, 167, 816.

