# Supplementary material for: The Role of the Anion Insertion‐Extraction Reaction in Amorphous Carbon Thin Film Electrodes on the Vanadium(IV/V) Reaction Probed by Scanning Electrochemical Cell Microscopy
Source: Small. 2025 Oct 7;21(46):e07044. doi: 10.1002/smll.202507044 (PMC12632429; doi:10.1002/smll.202507044)
Supplement: Supplementary file 1 — Supporting Information [file SMLL-21-e07044-s001.docx]

Supporting Information

**The role of the anion insertion-extraction reaction in amorphous carbon thin film electrodes on the vanadium(IV/V) reaction probed by scanning electrochemical cell microscopy**

*Maximilian Hamann^1^, Jens Carthäuser^1^, Diana Rata^2^, Nico Remmler^1^, Michael Bron^1^ and Matthias Steimecke^1*^*

^1^ Martin-Luther-Universität Halle Wittenberg, Institut für Chemie, Technische Chemie I, Von-Danckelmann-Platz 4, 06120 Halle/Saale

^2^ Martin-Luther-Universität Halle Wittenberg, Institut für Physik, Von-Danckelmann-Platz 3, 06120 Halle/Saale

*matthias.steimecke@chemie.uni-halle.de

**Sample characterization**


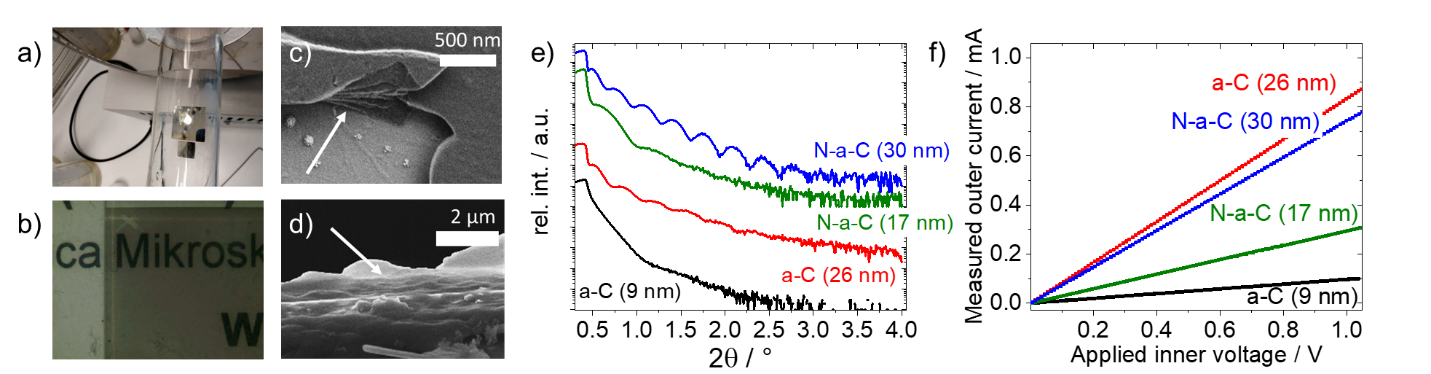


**Figure S1** a) Photos of a sample after removing from the oven and b) in an arrangement that shows its semitransparency, c) SEM of a scratch at a ~80 nm a-C sample, d) side view of a broken N-a-C sample with ~100 nm thickness (the arrows indicate positions where the layered structure is clearly visible), e) the results of the X-ray reflectivity measurements as well as f) the results of the four-point-probing to determine the sheet resistance of the four selected samples.

Figure S1a and b show a representative sample after synthesis with mirror-like appearance and semitransparency. The morphological structure can be analyzed when evaluating scratches or at the side view of a broken sample. The carbon films show a layered structure (Arrows in Figure S1c and d) where individual layers are peeled off when it is mechanically delaminated from the quartz glass substrate. X-ray reflectivity (XRR) measurements were used to determine the thickness of the amorphous carbon layers with and without nitrogen doping. Evaluation was done by analyzing the maxima of the Kiessing oscillation using Bragg`s law in equation 1. Here the layer thickness d is

| $d= \frac{n \lambda}{2 (\sin\theta_{m}-\sin\theta_{m+n})}$ | (1) |
| --- | --- |

with n – number of peak maxima, λ – wavelength of radiation (Cu Kα1, λ = 1.54056 Å) and θ_m_ and θ_m+n_ – angle of the respective oscillation maxima. It should be noted that this oscillation effect in Figure S1e only occurs with a densely packed material.

**X-ray diffraction (XRD) and infrared (IR) spectroscopy**

**Figure S2** a) X-ray diffraction pattern and b) IR spectra of the a-C and N-a-C samples with approximately 50 – 70 nm thickness. The color code in a) is also valid for b).

**Scanning electron microscopy (SEM)**


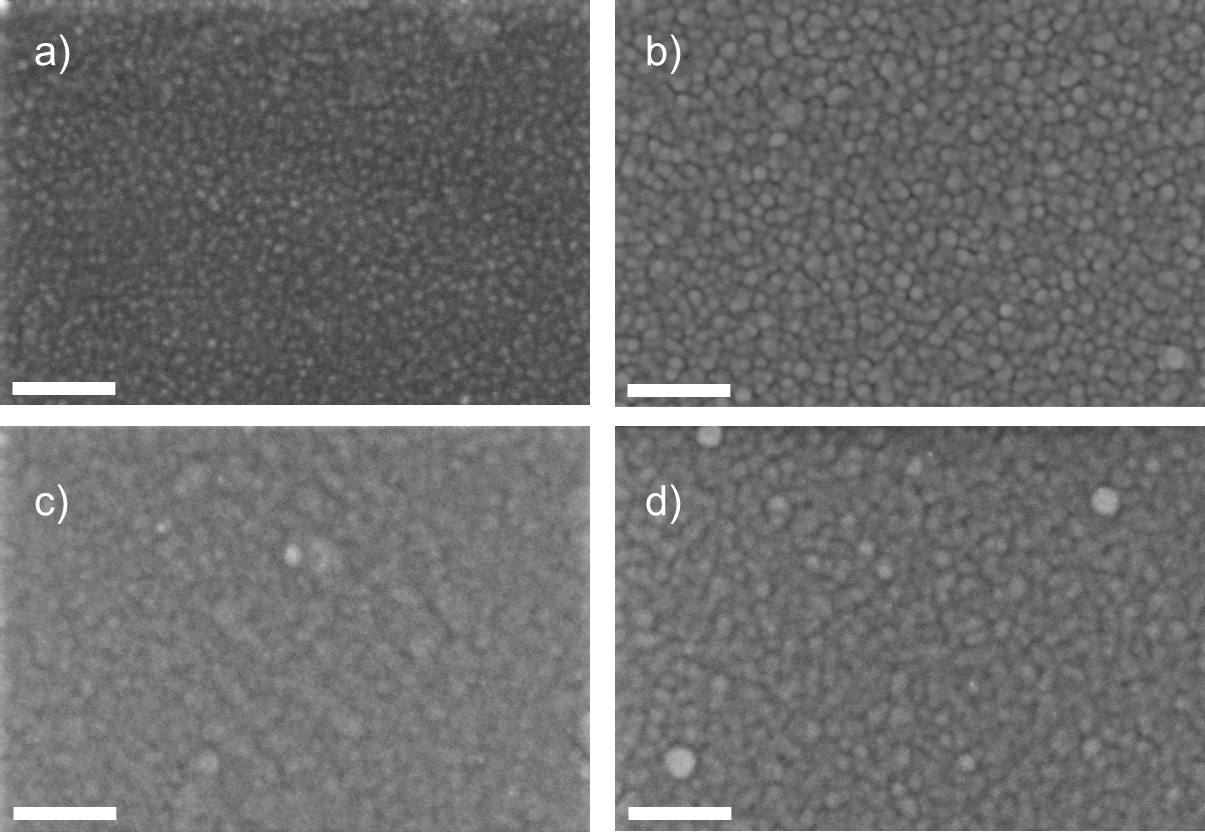


**Figure S3** Scanning electron microscopy images of the a) 9 nm a-C, b) 17 nm N-a-C, c) 26 nm a-C and d) 30 nm N-a-C sample. The scale bar represents 200 nm.

**X-ray photoelectron spectroscopy (XPS)**

**Figure S4** C 1s detail spectrum of the a) 9 nm a-C, b) 17 nm N-a-C, c) 26 nm a-C and d) 30 nm N-a-C sample with the deconvolution of individual structural contributions.

**Figure S5** N 1s detail spectrum of the a) 9 nm a-C, b) 17 nm N-a-C, c) 26 nm a-C and d) 30 nm N-a-C sample with the deconvolution of individual structural contributions.

**Figure S6** O 1s detail spectrum of the a) 9 nm a-C, b) 17 nm N-a-C, c) 26 nm a-C and d) 30 nm N-a-C sample with the deconvolution of individual structural contributions.

**Table S1** Summary of the best fits of the XPS detail scans from Figures S4, S5 and S6 for all samples with position of the respective peak, its full width at half maximum (FWHM) and the area.

|  | **a-C (9 nm)** | | | **a-C (26 nm)** | | |
| --- | --- | --- | --- | --- | --- | --- |
| **Component** | **Position / eV** | **FWHM** | **Area** | **Position / eV** | **FWHM** | **Area** |
| **O 1s** |  |  |  |  |  |  |
| C-O (sp^2^), C-O-C | 532.0 | 1.61 | 45418 | 532.0 | 2.88 | 21405 |
| C-O (sp^3^), C-OH | 532.8 | 1.77 | 88904 | 532.8 | 3.17 | 11156 |
| chemisorbed H_2_O | 535.2 | 1.52 | 3546 | 535.6 | 4.00 | 5725 |
| **C 1s** |  |  |  |  |  |  |
| C-C (sp^2^) | 284.0 | 1.41 | 170071 | 284.0 | 1.43 | 237716 |
| C-C (sp^3^) | 285.0 | 1.55 | 31027 | 285.0 | 1.57 | 32156 |
| C-O, C-OH, C-O-C | 286.1 | 2.12 | 32520 | 286.1 | 2.14 | 40614 |
| C=O | 286.9 | 1.41 | 2036 | 286.9 | 1.43 | 2388 |
| -O-C=O | 288.6 | 2.12 | 11929 | 288.6 | 2.14 | 13787 |
| carbonate | 290.0 | 2.12 | 5640 | 290.0 | 2.14 | 8018 |
| pi-pi* | 291.5 | 5.65 | 12494 | 291.5 | 5.71 | 18809 |
|  |  |  |  |  |  |  |
|  | **N-a-C (17 nm)** | | | **N-a-C (30 nm)** | | |
|  | **Position / eV** | **FWHM** | **Area** | **Position / eV** | **FWHM** | **Area** |
| **O 1s** |  |  |  |  |  |  |
| C-O (sp^2^), C-O-C | 532.0 | 2.39 | 33742 | 531.4 | 2.95 | 1861 |
| C-O (sp^3^), C-OH | 532.8 | 2.63 | 25658 | 532.2 | 3.24 | 19587 |
| chemisorbed H_2_O | 535.6 | 0.98 | 220 | 535.6 | 4.00 | 0.00 |
| **C 1s** |  |  |  |  |  |  |
| C-C (sp^2^) | 284.3 | 1.59 | 201760 | 284.3 | 1.59 | 174630 |
| C-C (sp^3^), C(sp^2^)-N | 285.3 | 1.75 | 46262 | 285.3 | 1.75 | 33929 |
| C-O, C-N=O, C(sp^3^)-N | 286.4 | 2.38 | 48389 | 286.4 | 2.39 | 40014 |
| C=O, O=C-N | 287.2 | 1.59 | 1018 | 287.2 | 1.59 | 0.00 |
| -O-C=O | 288.9 | 2.38 | 14379 | 288.9 | 2.39 | 11327 |
| carbonate | 290.3 | 2.38 | 6704 | 290.3 | 2.39 | 6226 |
| pi-pi* | 291.8 | 6.35 | 15041 | 291.8 | 6.36 | 14244 |
| **N 1s** |  |  |  |  |  |  |
| pyridinic | 398.0 | 2.00 | 12219 | 398.0 | 2.00 | 10129 |
| pyrrolic | 400.6 | 1.60 | 21678 | 400.6 | 1.60 | 18416 |
| quarternary | 401.6 | 1.00 | 3218 | 401.7 | 1.05 | 3392 |
| graphitic | 402.5 | 1.59 | 5925 | 402.7 | 1.21 | 2815 |
| N-Oxides/Nitro | 404.0 | 1.60 | 2741 | 404.0 | 1.60 | 2001 |

**Raman microscopy evaluation**

**Figure S7** Evaluation of the mean Raman spectra (N = 10) taken from Figure 1c with the deconvolution of individual band contributions of a) the 9 nm a-C, b) the 17 nm N-a-C as well as the c) the 26 nm a-C and d) the 30 nm N-a-C sample.

**Table S2** Summary of the best fits of the mean Raman spectra from Figure S7 for all samples with position of the respective band, its full width at half maximum (FWHM) and the area.

|  | **a-C (9 nm)** | | | **a-C (26 nm)** | | |
| --- | --- | --- | --- | --- | --- | --- |
| **Component** | **Position / cm^-1^** | **FWHM** | **Area** | **Position / cm^-1^** | **FWHM** | **Area** |
| I band | 1214.3 | 254.07 | 46.0876 | 1200.6 | 237.82 | 59.073 |
| D band | 1353.7 | 137.58 | 142.476 | 1356.1 | 156.80 | 140.193 |
| D3 band | 1524.0 | 194.63 | 58.1532 | 1524.7 | 207.59 | 69.8039 |
| G band | 1605.0 | 75.59 | 73.4432 | 1604.0 | 85.853 | 80.279 |
|  |  |  |  |  |  |  |
|  | **N-a-C (17 nm)** | | | **N-a-C (30 nm)** | | |
| **Component** | **Position / cm^-1^** | **FWHM** | **Area** | **Position / cm^-1^** | **FWHM** | **Area** |
| I band | 1191.8 | 233.21 | 48.536 | 1181.3 | 209.19 | 33.1497 |
| D band | 1373.1 | 201.28 | 156.43 | 1374.1 | 218.97 | 164.466 |
| D3 band | 1560.7 | 171.85 | 70.146 | 1561.1 | 166.52 | 54.1916 |
| G band | 1607.1 | 86.46 | 56.4154 | 1606.7 | 93.84 | 56.4342 |

**SECCM probing, setup, reference potential and evaluation of standard rate constants**

Micropipettes with openings <1 µm were equipped with a Platinum wire as a quasi-reference counter electrode (Pt-QRCE) in equimolar vanadium(IV/V) solution. The conversion at the opening of the capillary is limited in contrast to the dimension of the wire in solution (3-4 cm) so that the potential is constant during CV probing. Relevant iR drop was only observed at currents >10 nA. It was found that the Pt|V^(IV/V)^ half-cell accounts for a constant potential in 3 M H_2_SO_4_ electrolyte. The potential was determined against the reversible hydrogen electrode (Flexi RHE, Gaskatel) as follows:

**E(Pt|V^(IV/V^)** **= 1080 mV vs. RHE**

In contrast, the potential of a half-cell of 3 M H_2_SO_4_ without vanadium ions was found to be:

**E(Pt|3 M H_2_SO_4_) = 820 vs. RHE**

In case of the experiments with solely 3 M H_2_SO_4_ (Figure 5), the potential was calculated to the Pt|V^(IV/V)^-QRCE.


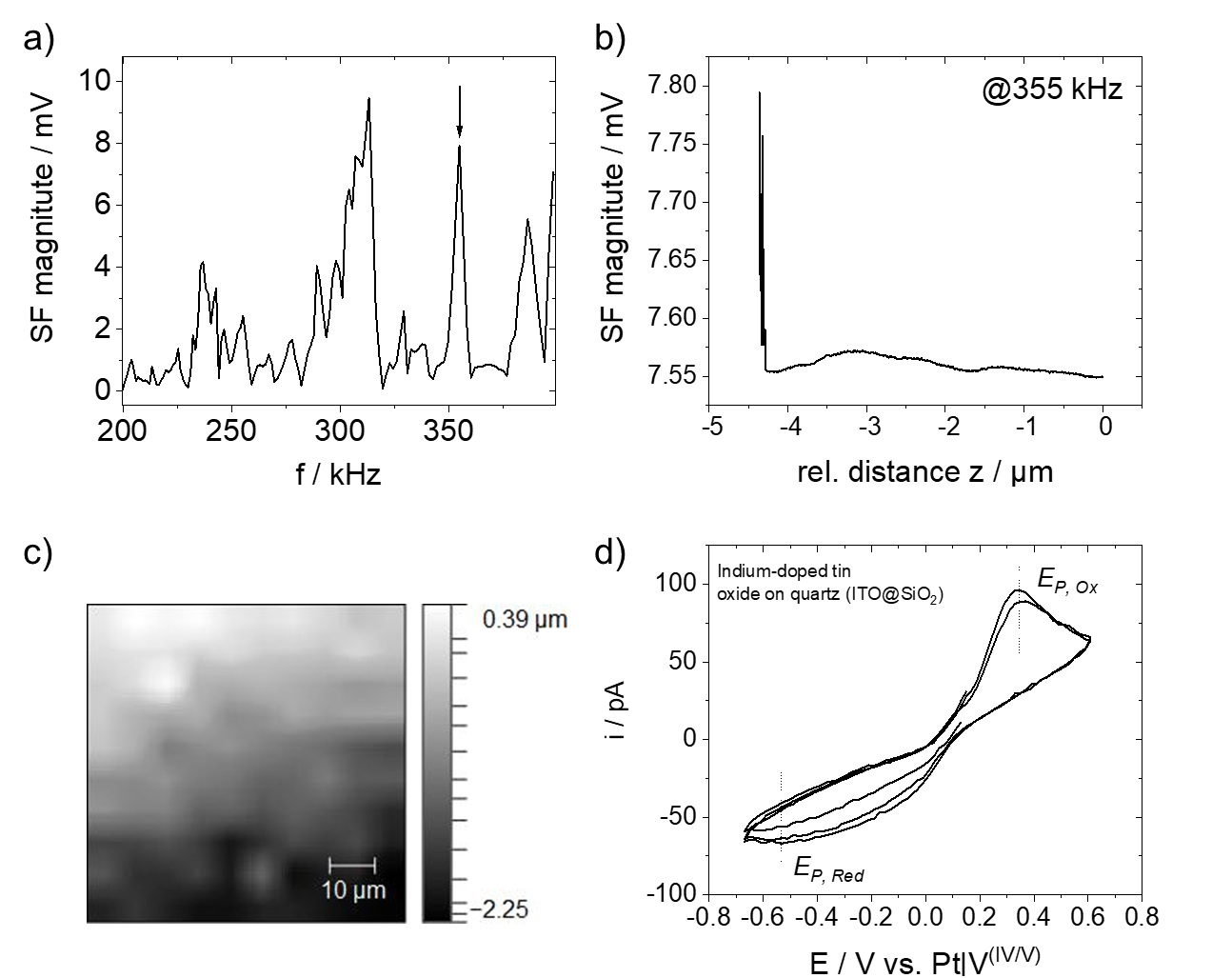


**Figure S8** a) Frequency spectrum of the setup shown in Figure 2a, b) shear force (SF) magnitude at 355 kHz during the approach curve, c) surface topography of a tilted sample and d) CV of Indium-doped tin oxide (ITO) reference sample.

The micropipette was equipped with two piezo actuators, which can be used for shear force microscopy technique. A typical frequency spectrum of the assembled probe is shown in Figure S8a. One of the strongest frequencies can be used to approach the micropipette, because the vibration is damped or enhanced by touching the surface. Here, the 355 kHz vibration was enhanced when the micropipette touched the samples surface (Figure S8b). By this proceeding, a surface topography (Figure S8c) was obtained and the sample tilt was calculated (~2 %). Additionally, Indium-doped tin oxide reference sample as a prominent non-carbon electrode material was probed by this method and a representative CV is displayed in Figure S8d.

**Degradation studies and ex situ Raman microscopy**


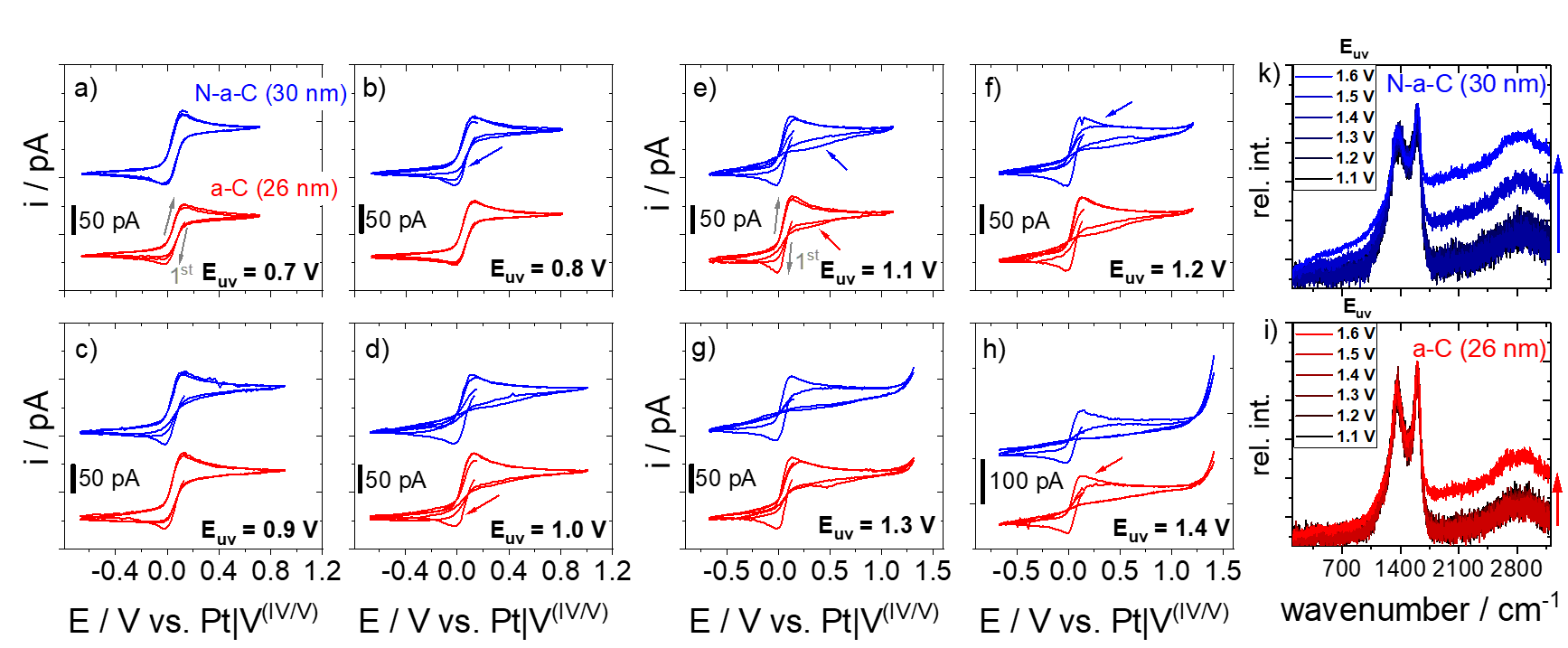


**Figure S9** Three consecutive cyclic voltammograms of the 26 nm a-C and the 30 nm N-a-C sample with increased upper vertex potential (E_uv_) of a) 0.7 V, b) 0.8 V, c) 0.9 V and d) 1.0 V for the low overpotential region and e) 1.1 V, f) 1.2 V, g) 1.3 V and 1.4 V for the high overpotential region as well as correlated ex situ Raman spectra of the very same SECCM spots of the i) 26 nm a-C and k) 30 nm N-a-C sample.

**Figure S10** Consecutive cyclic voltammograms of the a) 9 nm and b) 26 nm a-C samples as well as the c) 17 nm and d) 30 nm N-a-C samples with the upper vertex potentials (E_uv_) from 0.8 to 1.2 V. For better clarity only the first full and the half of the second CV is displayed. The scale bar in a) is also valid for b) – d) and a line (I = 0 A) is added to all diagrams.
